# Efficacy and safety of warm acupuncture in oxaliplatin-induced chronic peripheral neuropathy: a multicentre, randomized controlled trial protocol

**DOI:** 10.3389/fneur.2026.1824919

**Published:** 2026-07-29

**Authors:** Chenchen Li, Yi Ji, Xiaoqian Wu, Qinghua Shao, Guoli Wei, Jianping Liu, Jiege Huo

**Affiliations:** 1 Affiliated Hospital of Integrated Traditional Chinese and Western Medicine, Nanjing University of Chinese Medicine, Nanjing, Jiangsu, China; 2 The Third Clinical Medical College, Nanjing University of Chinese Medicine, Nanjing, Jiangsu, China; 3 The First Affiliated Hospital of Henan University of Chinese Medicine, Zhengzhou, Henan, China; 4 Center for Evidence-Based Medicine, Hospital of Chengdu University of Traditional Chinese Medicine, Chengdu, Sichuan, China; 5 Centre for Evidence-Based Chinese Medicine, Beijing University of Chinese Medicine, Beijing, China

**Keywords:** clinical research protocol, neurotoxicity grading, oxaliplatin-induced chronic peripheral neuropathy, randomized controlled trial, warm acupuncture

## Abstract

**Introduction:**

Oxaliplatin-induced chronic peripheral neuropathy (OICPN) is a prevalent side effect experienced by patients receiving chemotherapy that primarily manifests as ongoing numbness in the limbs that can persist after treatment ends. This condition significantly diminishes quality of life and frequently leads to dose reduction or early treatment cessation, compromising antitumor efficacy. However, a universally accepted and effective approach for the prevention or treatment of OICPN is currently unavailable. In our clinical observations, we have noted that warm acupuncture may relieve the symptoms of patients experiencing OICPN. Consequently, we propose this randomized controlled trial (RCT) to rigorously assess the safety and effectiveness of warm acupuncture for OICPN patients with the goal of providing preliminary evidence for its use in clinical settings.

**Methods and analysis:**

This study is a multicentre, randomized, controlled clinical trial involving 84 patients diagnosed with OICPN. Participants will be randomly divided into two groups in a 1:1 ratio to receive either standard care alone or warm acupuncture plus standard care. This regimen will include 16 sessions, with treatments occurring twice a week for the first month and once a week for the subsequent 2 months. All participants will complete the 12-week program and one-year follow-up. Outcomes will be assessed by blinded evaluators. The primary outcome is neurotoxicity graded by the National Cancer Institute Common Terminology Criteria for Adverse Events (NCI-CTCAE) version 4.0. Secondary outcomes include the European Organization for Research and Treatment of Cancer Quality of Life Questionnaire–Chemotherapy-Induced Peripheral Neuropathy 20-item scale (EORTC QLQ-CIPN20) and the European Organization for Research and Treatment of Cancer Quality of Life Core 30 Questionnaire (EORTC QLQ-C30), measured at baseline, week 2, month 1, post-treatment, and during follow-up.

**Clinical trial registration:**

https://www.chictr.org.cn/index.html, ChiCTR2400082506.

## 1 Background

Oxaliplatin is a platinum-based chemotherapy drug from the third generation that is commonly used to treat different types of solid tumors (1). FOLFOX chemotherapy, which pairs 5-fluorouracil with oxaliplatin, along with the XELOX chemotherapy, which combines capecitabine and oxaliplatin, are recognized as primary standard treatment options for colorectal cancer (2). The main adverse effects of oxaliplatin include peripheral neuropathy, myelosuppression, and gastrointestinal reactions (3).

Oxaliplatin-induced chronic peripheral neuropathy (OICPN) is a significant dose-limiting side effect that can hinder some patients’ ability to adhere to their treatment plans, often leading to reduced dosages or the complete cessation of chemotherapy, which in turn diminishes its effectiveness in clinical settings (4, 5).

Chronic peripheral neuropathy resulting from oxaliplatin affects approximately 40% of individuals undergoing treatment (6). This condition typically manifests as bilateral numbness and pain in a glove-and-stocking pattern, along with slight difficulties in fine motor skills and sensory ataxia. It follows a cumulative, dose-dependent trajectory, generally emerging and intensifying after approximately 3 months of ongoing therapy. Neuropathy classified as Grade 3 is observed in 50% of patients who have received a total dose of 1,170 mg/m^2^ (7). This condition often persists and may worsen even after treatment has ended, a phenomenon known as coasting (8). Research indicates that nearly 60% of patients report ongoing neuropathic symptoms following their last chemotherapy session, with 19% experiencing severe symptoms 18 months later (9). Additionally, 31% of patients continue to endure OICPN 5 years after completing chemotherapy (10). These symptoms significantly diminish the quality of life of cancer survivors.

Presently, therapeutic alternatives for antineoplastic drug-induced peripheral neuropathy are lacking in contemporary medicine. While clinical guidelines tentatively endorse duloxetine to alleviate neuropathic pain caused by OICPN, its use may lead to side effects such as fatigue, insomnia, and nausea (11–14). Additionally, the potential for drug interactions with various medications, including selective serotonin reuptake inhibitors, cytochrome P450 2D6 substrates, warfarin, and tamoxifen, has been recognized (15–18). In clinical settings, non-drug methods for managing OICPN mainly consist of acupuncture and functional exercises (11). These techniques are noted for their ease of use, safety, and patient acceptance. Nonetheless, the effectiveness of these treatments is backed by evidence from medical research, including studies on surgical procedures for spontaneous intracerebral hemorrhage and online brief behavioral therapy for insomnia. In the future, well-designed randomized controlled trials are essential to better define the effectiveness and clinical relevance of different treatment options for OICPN.

Acupuncture is a widely practiced and safe treatment option that is essential to address peripheral neuropathy. In the last 10 years, a significant number of RCTs on acupuncture for chemotherapy-induced peripheral neuropathy have been conducted both in China and abroad. Recent research and reviews indicate that acupuncture is an effective and safe approach for alleviating neurotoxicity caused by chemotherapy, with few side effects. A range of acupuncture techniques, such as manual acupuncture (19–23), electroacupuncture (24–27), moxibustion (28, 29), warm acupuncture (30–33), and acupoint injection (34), have generated positive outcomes, whether used alone or alongside other therapies (25).

Warm acupuncture integrates traditional acupuncture with moxibustion by igniting moxa wool placed on the handle of the needle. The heat generated from the burning moxa travels through the needle to stimulate specific acupoints. This method is known to alleviate cold, clear blockages, boost yang energy, enhance overall qi, and improve blood flow. Additionally, the heat and distinctive volatile substances released during moxa burning are thought to improve microcirculation and bodily functions, stimulate various systems, and increase tissue metabolism and heat generation. Earlier research has suggested the potential benefits of warm acupuncture for OICPN (30–33). However, these investigations often suffer from methodological flaws, including being conducted at a single centre (30, 31). Common issues in previous studies include small participant groups, brief follow-up periods, varying outcome measures, and a lack of standardized procedures (30–33). We have developed a multicentre, randomized controlled trial for colorectal cancer patients to overcome these challenges. This trial will include multiple follow-up visits over a comprehensive 12-month timeframe, implement a rigorously standardized warm acupuncture protocol, and utilize internationally accepted neurotoxicity grading scales and quality-of-life questionnaires for evaluation. The goal of this research is to deliver empirical evidence on the effectiveness of warm acupuncture in managing oxaliplatin-induced neurotoxicity, supported by both clinical findings and theoretical insights.

## 2 Methods and analysis

### 2.1 Study design

This clinical trial is designed as a randomized, controlled study with the assessors blinded to the treatment allocation. It will be conducted in six medical facilities throughout China, including the Jiangsu Province Hospital with Integration of Chinese and Western Medicine, Huai’an Hospital of Traditional Chinese Medicine, Yancheng Hospital of Traditional Chinese Medicine, Jiangyin Hospital of Traditional Chinese Medicine, Dongtai Hospital of Chinese Medicine, and Nanjing Lishui District Hospital of Traditional Chinese Medicine. The recruitment of participants will occur simultaneously in outpatient settings and hospital wards.

The Institutional Ethics Committee at the coordinating centre has reviewed and granted approval for this study, as indicated by the approval number 2024-LWKYZ-008, which remains valid until February 23, 2027. Prior to participation, all individuals will provide written informed consent. Participants who are enrolled will receive complimentary warm acupuncture treatment. The methodology in this trial is meticulously organized, ensuring that ethical standards are met. The protocol aligns with the Standard Protocol Items: Recommendations for Interventional Trials (SPIRIT) guidelines to uphold scientific integrity (35). Additionally, the results will be reported in accordance with the relevant Consolidated Standards of Reporting Trials (CONSORT) extension guidelines, ensuring rigorous quality control throughout the research (36).

**Figure 1:**
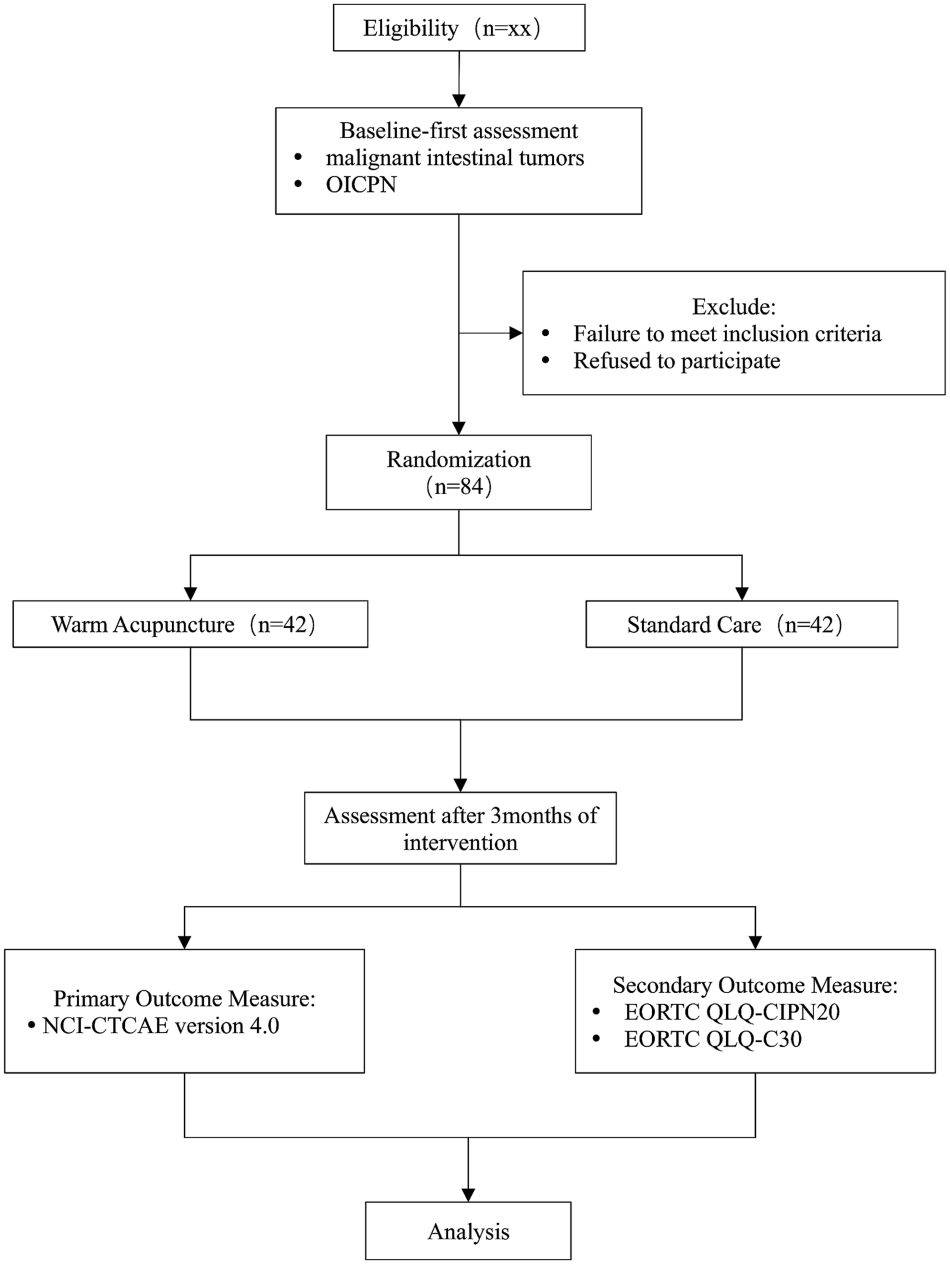
Flow diagram of this study. A total of 84 participants will be included in this study and will be randomized (1:1) to two groups: (1) A standard care group with only typical protective guidelines and (2) a warm acupuncture group with the same standard care and a 12-week warm acupuncture regimen. All participants will complete the 12-week intervention and a subsequent one-year follow-up.

Participants who meet the criteria will be randomly divided into two groups, with an equal ratio of 1:1, assigning them to either the warm acupuncture group or the standard care group. All individuals will participate in a treatment phase lasting 12 weeks, followed by a follow-up period of 1 year. Medical statisticians will analyse the outcome data. This trial has been officially registered with the Chinese Clinical Trial Registry (Registration number: ChiCTR2400082506). The flow of this study is illustrated in Figure 1, while a comprehensive schedule for enrolment, interventions, and assessments is provided in Table 1.

**Table 1:** Schedule of enrolment, interventions, and assessments.

Study Schedule	Study period								
	Baseline	Discharge				Follow-up			
Timepoint	T0	T1	T2	T3	T4	T5	T6	T7	T8
Week	0	2	4	8	12	16	24	36	60
Enrollment									
Eligibility screen	**×**								
Informed consent	**×**								
Demographics	**×**								
Medical history	**×**								
Physical examination	**×**								
Laboratory examination	**×**								
CT/MRI	**×**								
ECG	**×**								
Neurological evaluation	**×**								
Interventions									
Standard care group		3 months of treatment							
Warm acupuncture group		3 months of treatment							
Assessment									
NCI-CTCAE V4.0	**×**	**×**	**×**	**×**	**×**	**×**	**×**	**×**	**×**
EORTC QLQ-CIPN20	**×**	**×**	**×**	**×**	**×**	**×**	**×**	**×**	**×**
EORTC QLQ-C30	**×**	**×**	**×**	**×**	**×**	**×**	**×**	**×**	**×**

### 2.2 Inclusion criteria

Participants who met all of the following requirements were considered for inclusion: (1) Aged 18–85 years; (2) Completion of chemotherapy (at least 3 months prior) using the mFOLFOX6 or XELOX regimen*; (3) Presence of chemotherapy-induced peripheral neuropathy with a neurotoxicity grade ≥2 according to the NCI-CTCAE version 4.0; (4) Absence of severe cardiac, hepatic, renal, or hematopoietic system impairments; (5) Karnofsky Performance Status (KPS) score ≥60 and an expected survival of ≥6 months; (6) Absence of any cognitive or mental health issues; possesses typical verbal skills to effectively articulate symptoms and the overall state; (7) Provision of written informed consent.


$$\begin{array}{l} {}*\text{mFOLFOX}6—\text{oxaliplatin},\text{leucovorin},\text{fluorouracil};\\ \text{XELOX}—\text{oxaliplatin},\text{capecitabine} \end{array}$$

### 2.3 Exclusion criteria

Patients were excluded if they had: (1) A record of other cancerous tumors in the last 5 years, excluding those with carcinoma *in situ* that has been completely treated; (2) Presence of other neurological disorders (e.g., diabetic neuropathy and infectious or toxic neuropathy); (3) Current use of other medications with known neurotoxicity; (4) Medications are currently being utilized for the prevention and management of neuropathic pain, including duloxetine, gabapentin, pregabalin, venlafaxine, and mecobalamin; (5) Pregnancy, planned pregnancy, or lactation; (6) Presence of psychiatric or consciousness disorders impairing cooperation; (7) Severe skin diseases or ulceration occurring on the limbs; (8) Having received acupuncture treatment for any indication within the past 1 month; (9) Currently participating in any other clinical trial of a drug.

### 2.4 Sample size calculation

This study employs a randomized controlled trial methodology to assess the effectiveness of warm acupuncture for alleviating peripheral neuropathy caused by oxaliplatin, comparing a warm acupuncture cohort with a standard care cohort. The main measure of interest is the neurotoxicity level, which will be evaluated using the NCI-CTCAE version 4.0 criteria. Based on previous research, a treatment response is indicated by a decrease of one or more grades in neurotoxicity (37). The expected response rates for the two groups are 0.8 and 0.47, respectively. Utilizing a standard formula for the comparison of two proportions, with a two-tailed alpha level of 0.05 and a power of 80% (*β* = 0.2), a minimum of 33 participants is necessary for each group. Considering an estimated dropout rate of approximately 20%, the target sample size is established at no less than 40 participants per group. Consequently, the overall sample size for this investigation is set at 84 participants, with 42 in each group.

### 2.5 Randomization and allocation concealment

A statistician who is not part of the clinical trial will create the randomization sequence utilizing SAS software, version 9.4 (SAS Institute Inc., Cary, NC, United States). This sequence will utilize a block randomization approach with block sizes of 4, 6, or 8, organized by study centre. The resulting randomization list will then be uploaded to an Interactive Web Response System (IWRS). After screening an eligible participant and obtaining informed consent, the site investigator will access the IWRS to enter the participant’s basic identifiers along with the centre code. The system will then automatically generate a unique randomization number and assign the appropriate treatment group, ensuring that allocation remains fully concealed. Regular audits of IWRS procedures and documentation will be conducted to ensure consistent implementation of the allocation workflow across all centers.

### 2.6 Data collection and monitoring

This study will employ a uniform electronic data capture (EDC) platform for managing data. All site research coordinators will receive centralized training on uniform screening procedures and case report form completion standards. Trained research coordinators will independently enter all information from the case report forms into the EDC system. The platform will automatically conduct logic and range validations. Source data verification will follow a set monitoring strategy, with clinical research associates conducting frequent on-site inspections at each participating site to maintain operational uniformity and confirm the alignment between the electronic data and the original medical records.

To further ensure treatment fidelity, these on-site inspections will include direct observation of warm acupuncture sessions to verify protocol adherence. In addition, a detailed Standard Operating Procedure (SOP) manual has been developed for this trial to specify all operational parameters and minimize inter-practitioner variability. Prior to trial initiation, all acupuncturists will receive centralized training on the SOP and will be certified only after demonstrating consistent competency in performing the standardized warm acupuncture procedures. These on-site inspections will also monitor randomization and enrollment procedures to ensure consistent implementation of the IWRS-based allocation workflow across all centers.

The Jiangsu Province Hospital with Integration of Chinese and Western Medicine will oversee this trial. An independent Data Monitoring Committee (DMC) will be formed to uphold the integrity and safety of this trial. This committee will regularly assess the gathered data and review the safety and efficacy outcomes of the research. Comprising specialists in oncology, statistics, and research methods, the committee will have the authority to request unblinding during both interim and final evaluations. Any serious adverse events must be reported to the principal investigator, the ethics committee, and the sponsor within a 24-h timeframe, while ongoing safety monitoring will be facilitated through the EDC system.

### 2.7 Blinding

Given the unique characteristics of acupuncture techniques, employing a double-blind approach is impractical in this study. Both the practitioners providing the acupuncture and the patients receiving it will know which treatment they are assigned. The processes for administering the treatment and assessing the results will be distinctly divided to reduce any possible bias. Practitioners conducting the warm acupuncture will not be involved in the evaluation of the treatment results. During the course of the study, the individuals assessing outcomes and the statistician analyzing the data will not have knowledge of the treatment assignments.

## 3 Interventions

### 3.1 Standard care group

Participants in the standard care group will be instructed to: (1) Avoid contact with either cold or hot water; (2) Wear gloves and socks to protect their hands and feet; (3) Avoid direct contact with metal objects; (4) Refrain from alcohol consumption; (5) Avoid strenuous exercise such as long-distance hiking.

### 3.2 Warm acupuncture group

Alongside standard care, patients will receive warm acupuncture therapy. They will lie on their backs with the treatment areas properly exposed and sanitized. Acupuncture will be applied to the following specific points: Quchi (LI11), Waiguan (TE5), Hegu (LI4), and Baxie (EX-UE9) on the arms; Guanyuan (CV4) on the abdomen; and Zusanli (ST36), Sanyinjiao (SP6), Taichong (LR3), and Bafeng (EX-LE10) on the legs. A qualified practitioner with 3 years of experience will perform needling using single-use stainless steel needles (0.25 mm × 40 mm, sourced from Suzhou Hualun Medical Apparatus Co., Ltd., China). The insertion depth and angle will follow the guidelines in The Science of Acupuncture, utilizing a consistent reinforcing–reducing technique to prioritize patient safety and treatment efficacy. Once the deqi sensation is achieved, a 27-mm moxa stick (from Nanyang Baicaotang Natural Moxa Products Co., Ltd., made from 10-year aged moxa, measuring 18 mm × 27 mm) will be placed on the needle handles at Zusanli (ST36) and Guanyuan (CV4). The moxa stick will be held approximately 20 mm above the skin, separated by a small paper sheet. The base of the moxa stick will be ignited and allowed to burn out completely, after which the paper will be removed while the moxa remains on the needle. The needles will generally remain in place for 20 to 30 min before the ash is cleared and the needles are removed. This treatment will occur twice weekly during the first 4 weeks and once weekly from weeks five to 12, with each session lasting 30 min, completing a full 12-week course. This tapered frequency schedule was designed to achieve rapid symptom control during the initial intensive phase and to consolidate therapeutic effects during the subsequent maintenance phase.

### 3.3 Outcomes

Medical professionals will evaluate neurotoxicity at several key intervals: at the start of treatment; at 2 weeks, 4 weeks, and 8 weeks after treatment; and at 1 month, 3 months, 6 months, and 12 months after the initial treatment cycle. The assessment will utilize the NCI-CTCAE version 4.0, along with the EORTC QLQ-CIPN20 and EORTC QLQ-C30 scales. Safety will be monitored by recording any adverse events in accordance with the NCI-CTCAE version 4.0 guidelines.

#### 3.3.1 Primary outcome measure

##### 3.3.1.1 Neurotoxicity grading

Peripheral neurotoxicity will be assessed using the NCI-CTCAE version 4.0. This classification categorizes neuropathy into four distinct grades: Grade 1 indicates the presence of areflexia or paraesthesia that does not affect functionality; Grade 2 involves sensory changes or paraesthesia that disrupts function but does not impact daily living activities; Grade 3 refers to sensory changes or par-aesthesia that hinders daily living activities; and Grade 4 represents a severe sensory–motor neuropathy that greatly impairs daily living activities. To ensure the reliability of the CTCAE grading, all outcome assessors will receive standardized training on the CTCAE version 4.0 criteria prior to trial initiation, and periodic quality checks will be performed to minimize inter-evaluator variability. If discrepancies are identified, the grading will be reviewed and adjudicated by a designated specialist.

#### 3.3.2 Secondary outcome measures

##### 3.3.2.1 Neurotoxicity symptoms

These symptoms will be assessed using the EORTC QLQ-CIPN20. The intensity of chemotherapy-induced peripheral neuropathy is measured through the cumulative score obtained from the EORTC QLQ-CIPN20 questionnaire. This validated patient-reported outcome measure (PROM) is well-established for evaluating peripheral neuropathy, especially chemotherapy-induced peripheral neuropathy. Comprising 20 questions that address sensory, motor, and autonomic functions, the questionnaire utilizes a 4-point Likert scale, with scores ranging from 1 to 4. The total score can vary between 20 and 80, which is subsequently converted to a scale of 0 to 100. A higher score reflects a more severe level of neurotoxic symptoms.

##### 3.3.2.2 Severity of neuropathy and quality of life

These parameters will be measured using the EORTC QLQ-C30, a questionnaire developed by the European Organization for Research and Treatment of Cancer.

The EORTC QLQ-C30 is a prominent tool used globally to evaluate quality of life in oncology clinical research (38). It includes five functional dimensions, physical, role, cognitive, emotional, and social quality of life, along with three symptom dimensions (fatigue, pain, and nausea/vomiting), six individual symptom assessments (dyspnoea, appetite loss, insomnia, constipation, diarrhea, and financial issues), and a global health status measure. Higher scores in the functional dimensions, which assess aspects such as balance and daily activities, reflect an improved quality of life, similar to what is measured by the Fugl–Meyer and Berg balance scales. In contrast, higher scores in the symptom dimensions and individual assessments, such as those related to pain or depression, indicate a decline in quality of life or a greater symptom burden.

#### 3.3.3 Safety assessment

The evaluation of adverse events will be conducted using the NCI-CTCAE version 4.0, released on June 14, 2010. This terminology serves as a framework for reporting adverse events. Following each treatment session, an additional safety assessment will be performed to record any side effects experienced by participants in both treatment groups.

## 4 Data analysis

### 4.1 Statistical analysis sets

Full Analysis Set (Intention-to-Treat): All randomized participants will be included in the full analysis set and analyzed based on their initial randomized group assignment, irrespective of their compliance with the full intervention protocol. This set will constitute the primary population for the efficacy analysis.

Per-Protocol Set: This set will consist of participants who complete at least 80% of the planned treatment sessions (i.e., at least 13 sessions over the 12-week period) and have no major protocol deviations. It will be utilized for supportive sensitivity analyses.

Safety Analysis Set: This set shall encompass all participants who undergo at least one session of warm acupuncture and will be employed for the safety assessment of the intervention.

### 4.2 Statistical methods

Analysis of Baseline Characteristics: The baseline characteristics of each group will be delineated using appropriate summary statistics. For normally distributed continuous variables, the results will be shown as means ± standard deviations, with independent sample t-tests utilized for comparisons between groups. If the distribution is not normal, these variables will be reported as medians (interquartile ranges) and analyzed using the Mann–Whitney U test. Categorical data will be reported as frequencies (percentages) and analyzed for differences between groups using either the Chi-square test or Fisher’s exact test, depending on the situation.

Analysis of the Primary Outcome: The primary focus will be on the ordinal categorical variable representing the neurotoxicity grade according to NCI-CTCAE version 4.0. A Generalized Estimating Equations (GEE) approach or a mixed-effects ordinal logistic regression model will be utilized to analyse the primary outcome. This model will be used to conduct a statistical assessment comparing the effectiveness of warm acupuncture with alternative treatments, emphasizing differences between groups, temporal effects, and their interactions to determine its role in reducing neurotoxicity.

Analysis of the Secondary Outcomes: Secondary outcomes, which include the continuously measured scores from the EORTC QLQ-CIPN20 and the commonly used EORTC QLQ-C30 scales, will be analyzed using a linear mixed-effects model. This model will incorporate fixed effects for group, time, visit, and the interaction between group and time, while subject will be treated as a random effect. This approach aims to assess the variations in symptom burden and quality of life across groups and their changes over time.

Additional statistical details are predefined. In the primary models, we will adjust for key baseline covariates that display an imbalance (*p* < 0.1) or possess established prognostic significance (e.g., number of chemotherapy cycles). If a significant group-by-time interaction is identified, Bonferroni-corrected *post hoc* pairwise comparisons will be conducted. Missing data, assumed to be missing at random, will be managed using the likelihood-based estimation inherent to linear mixed models; the robustness of findings will be assessed through sensitivity analyses, including the last observation carried forward method. All adverse events will be summarized using descriptive statistics, and between-group differences in incidence rates will be evaluated using the Chi-square test or Fisher’s exact test, as appropriate. For participants who withdraw from the study, the primary efficacy analysis will follow the Intention-to-Treat (ITT) principle, with linear mixed models handling missing data under the Missing at Random assumption. A Per-Protocol (PP) analysis will be conducted as a sensitivity measure. All withdrawals and protocol deviations will be recorded with detailed reasons.

## 5 Discussion

OICPN is a frequent side effect of cancer chemotherapy that is mainly characterized by sensations of numbness, discomfort, and sensorimotor issues in the extremities, which significantly diminishes patients’ quality of life and their ability to adhere to treatment. At present, effective treatments for OICPN are scarce in clinical settings. Standard care, which primarily emphasizes health education and managing symptoms, shows limited effectiveness. Acupuncture, a treatment approach rooted in traditional Chinese medicine, has distinct benefits in addressing peripheral neuropathy. Recent research and clinical trials indicate that acupuncture and other complementary therapies may effectively reduce OICPN symptoms (19–23). Among the various acupuncture methods, warm acupuncture combines the physical stimulation from needling with the heat generated by moxibustion. This added warmth is believed to increase the flow of qi and blood within the meridians, aligning with the views of traditional Chinese medicine on the underlying causes of cold-related issues in individuals with OICPN and blood stagnation. Nonetheless, existing clinical investigations into warm acupuncture for OICPN often face limitations such as being conducted at single centres, involving small participant groups, having brief follow-up durations, inconsistent outcome measures, and inadequate procedural standardization descriptions (30–33). Consequently, the clinical effectiveness and long-term advantages of warm acupuncture therapy require validation through well-structured, multicentre randomized controlled trials, akin to the current research.

This study utilizes a multicentre, randomized controlled approach to apply a standardized warm acupuncture treatment in conjunction with standard care. The choice of acupoints is based on principles from The Science of Acupuncture, clinical experience, and data mining studies that identify frequently utilized point patterns for chemotherapy-induced peripheral neuropathy (39, 40). The selection process incorporates both local and distal point combinations. For the upper limbs, points like Quchi (LI11) and Waiguan (TE5) were chosen to facilitate meridian flow, while lower limb points such as Zusanli (ST36) and Sanyinjiao (SP6) were selected to balance qi and blood. Guanyuan (CV4) was included to strengthen the foundation and stabilize the source. The focused use of warm acupuncture at Guanyuan (CV4) and Zusanli (ST36) merges the therapeutic effects of acupuncture with the warming and circulation-boosting properties of moxibustion. The heat from the burning moxa penetrates deeper tissues through the needle, enhancing therapeutic effects that involve warming yang, boosting qi, and nourishing the meridians, thus creating a synergistic relationship between needling and moxibustion. From a neurobiological perspective, the therapeutic effects of warm acupuncture on CIPN may be mediated through multiple mechanisms that directly address the key pathophysiological processes of this condition. The thermal and mechanical stimulation generated by warm acupuncture can improve local microcirculation and increase blood supply to peripheral nerves, thereby counteracting the ischemic and metabolic disturbances that contribute to axonal degeneration. In addition, acupuncture-induced afferent input has been shown to activate descending serotonergic and noradrenergic inhibitory pathways, which suppress spinal nociceptive transmission—a mechanism similar to that of duloxetine, the currently recommended pharmacotherapy for CIPN (41). Preclinical evidence also suggests that acupuncture can reduce neuroinflammation by attenuating spinal glial cell activation and the release of pro-inflammatory cytokines (TNF-*α*, IL-1β, IL-6) that sensitize peripheral nociceptors (42, 43), and may modulate ion channel function, including voltage-gated sodium channels and TRPA1 channels, thereby reducing neuronal hyperexcitability and cold hypersensitivity (44, 45). Furthermore, by promoting mitochondrial function and enhancing neurotrophic support, warm acupuncture may facilitate axonal repair and regeneration (46, 47). While these mechanistic hypotheses require future validation, they provide a plausible neurobiological framework that complements the traditional Chinese medicine rationale for warm acupuncture in CIPN treatment. A consistent needling technique, moxa type, and treatment protocol were employed for all participants to address the variability that is often observed in acupuncture studies. Additionally, all procedures were conducted by certified acupuncturists to reduce the influence of practitioner differences on the results. Notably, this study included several follow-up assessments over a 12-month duration. This design facilitates the evaluation of both the immediate effects of warm acupuncture and a thorough investigation of its long-term effects on neurological recovery and improved quality of life. The outcome measures encompass both clinician-assessed scales and patient-reported outcomes, providing a comprehensive evaluation that spans from objective neurotoxicity assessments to subjective sensory experiences and improvements in quality of life. This approach aims to comprehensively assess the therapeutic outcomes of warm acupuncture.

This study has a number of limitations. First, the absence of a sham acupuncture control limits our ability to distinguish specific from non-specific effects; however, this pragmatic design was chosen to address the clinically relevant question of whether warm acupuncture provides additional benefit over standard care alone in real-world practice. Second, due to the distinct nature of acupuncture techniques, blinding of either the practitioners or the participants was not possible, which could introduce biases in performance and measurement. Blinded data assessment and analysis were employed in this study as a partial solution to improve objectivity.

The results of this study highlight considerable clinical significance, especially regarding the use of acupuncture in diverse medical disciplines. Should warm acupuncture be shown to effectively reduce symptoms of OICPN, it would equip healthcare providers with a reliable and practical non-pharmacological treatment approach. These data could guide future revisions of treatment protocols, integrating warm acupuncture into the current therapeutic landscape. Such integration would broaden clinical treatment alternatives and ultimately improve patients’ overall well-being. Even if positive outcomes are not obtained for the main objective, the established standardized methods and the long-term data gathered will serve as vital references for future initiatives aimed at refining the intervention strategy and determining the best timing for treatment. Regardless of the primary results, this meticulously executed study will set a methodological standard in warm acupuncture for OICPN, fostering the standardization and scientific integrity of future research. Future studies with rigorous sham acupuncture controls are warranted to confirm our findings.
